# Acute Rheumatic Fever Without Pharyngitis: A Case Report

**DOI:** 10.1002/jgf2.70163

**Published:** 2026-08-06

**Authors:** Hiroki Suzuyama, Ryutaro Hashimoto, Fumiya Taketomi, Ikuma Noge, Tadaaki Arimura, Hiroaki Takeoka, Atsuhiko Sakamoto, Shigeki Nabeshima

**Affiliations:** ^1^ Department of General Internal Medicine Fukuoka University Hospital Fukuoka Japan; ^2^ Department of Cardiology Fukuoka University Hospital Fukuoka Japan

**Keywords:** acute rheumatic fever, atrioventricular block, group A *Streptococcus*

## Abstract

An 18‐year‐old man presented with fatigue and polyarthralgia refractory to nonsteroidal anti‐inflammatory drugs (NSAIDs). He had no history of sore throat or other upper respiratory symptoms. Reactive arthritis was initially suspected. However, persistent symptoms, fever, polyarthritis, a cardiac murmur, atrioventricular block, and an elevated antistreptolysin O titer (739 IU/mL) suggested acute rheumatic fever. He met the revised Jones criteria for acute rheumatic fever. Treatment with amoxicillin and corticosteroids rapidly resolved his symptoms and electrocardiographic abnormalities.

## 1 Background

Acute rheumatic fever (ARF) is a rare inflammatory disease in developed countries, but it can cause serious cardiac sequelae if not diagnosed promptly. Although ARF typically follows group A *streptococcal* (GAS) pharyngitis, antecedent pharyngeal symptoms are often absent or difficult to recall at presentation. In primary care settings, ARF may therefore be overlooked, particularly in young patients presenting with nonspecific symptoms such as polyarthralgia or fatigue. We report a case of ARF without a history of pharyngitis, highlighting the diagnostic challenges and the importance of considering ARF in the differential diagnosis of unexplained carditis or arrhythmias in young adults.

## 2 Case Presentation

An 18‐year‐old man presented with fatigue and progressively spreading polyarthralgia involving the left knee, right knee, right elbow, and predominantly the proximal interphalangeal joints of the left hand. The symptoms were refractory to NSAIDs.

He had no history of pharyngitis or upper respiratory symptoms.

On initial examination, he was afebrile and hemodynamically stable. There was no pharyngeal erythema, tonsillar swelling, or heart murmur. Tenderness and warmth were present in multiple joints without obvious swelling, and no skin rash was observed. Laboratory testing showed a white blood cell count of 11,300/μL and a C‐reactive protein (CRP) level of 4.57 mg/dL. Reactive arthritis was initially suspected.

The patient had been self‐administering excessive diclofenac. After the dose was adjusted, he was followed as an outpatient. Five days later, worsening inflammatory markers (CRP, 8.22 mg/dL) and persistent joint symptoms prompted hospital admission for further evaluation. Laboratory findings are summarized in Table 1.

**TABLE 1:** Laboratory findings at the initial visit and 5 days after the initial visit.

	First visit	Day 5
WBC (/μL)	11,300	11,800
Neu (%)	71	74
Ly (%)	25	21
Mono (%)	3.7	3.5
RBC (×10^6^/μL)	4.01	4.49
MCV (%)	90	
Hb (g/dL)	12.1	13.4
Ht (%)	36.1	40.4
Plt (×10^4^/μL)	43.1	49.9
ESR	> 120	
TP (g/dL)	7.9 g	
Alb (g/dL)	3.8 g	
BUN (mg/dL)	13	
Cre (mg/dL)	0.96	
T‐Bil (mg/dL)	0.7	
Glu (mg/dL)	89	
AST (U/L)	36	77
ALT (U/L)	90	141
LDH (U/L)	283	225
γ‐GTP (U/L)	84	103
CRP (mg/dL)	4.57	8.22
Amy (U/L)	47	
CK (U/L)	759	75
Na (mmol/L)	141	
K (mmol/L)	4.5	
Cl (mmol/L)	104	

Abbreviations: Alb, albumin; ALT, alanine aminotransferase; Amy, amylase; AST, aspartate aminotransferase; BUN, blood urea nitrogen; CK, creatine kinase; Cl, chloride; Cre, creatinine; CRP, C‐reactive protein; ESR, erythrocyte sedimentation rate; Glu, glucose; GTP, gamma‐glutamyl transpeptidase; Hb, hemoglobin; Ht, hematocrit; K, potassium; LDH, lactate dehydrogenase; Ly, lymphocytes; MCV, mean corpuscular volume; Mono, monocytes; Na, sodium; Neu, neutrophils; Plt, platelet count; RBC, red blood cell count; T‐Bil, total bilirubin; TP, total protein; WBC, white blood cell count.

On admission, tenderness involved the left‐predominant PIP joints, both wrists, the right elbow, and both knees, with knee warmth but no swelling.

Temporary discontinuation of NSAIDs resulted in a fever of 39°C and worsening arthralgia. Although fever resolved after NSAIDs were resumed, arthralgia persisted.

On day 8 after the initial visit, the patient developed chest discomfort and a holosystolic murmur was noted. Electrocardiography revealed second‐degree atrioventricular block (Mobitz type II) with marked PR interval prolongation. Transthoracic echocardiography revealed mild aortic regurgitation (AR) and mitral regurgitation (MR). According to the 2023 World Heart Federation echocardiographic criteria, the patient fulfilled the criteria for pathological aortic regurgitation, corresponding to Stage A. Gallium scintigraphy demonstrated abnormal uptake corresponding to the painful joints. ARF was suspected based on polyarthritis, fever, PR prolongation, and valvular regurgitation.

Although the rapid antigen detection test and throat culture for GAS were negative, the anti‐streptolysin O (ASO) titer was elevated to 739 IU/mL, indicating recent GAS infection. The patient fulfilled the revised Jones criteria and was diagnosed with ARF. Oral amoxicillin (2000 mg/day) was initiated. Because of NSAIDs‐refractory polyarthralgia, Mobitz type II atrioventricular block, and valvular regurgitation, oral prednisolone was initiated at 60 mg/day (0.5 mg/kg/day). The patient's joint pain resolved completely by the following day. Electrocardiography showed resolution of Mobitz type II AV block, and PR prolongation gradually improved. Prednisolone was gradually tapered and discontinued. Because mild valvular regurgitation persisted, secondary prophylaxis with oral benzylpenicillin (400,000 units/day) was initiated.

**FIGURE 1:**
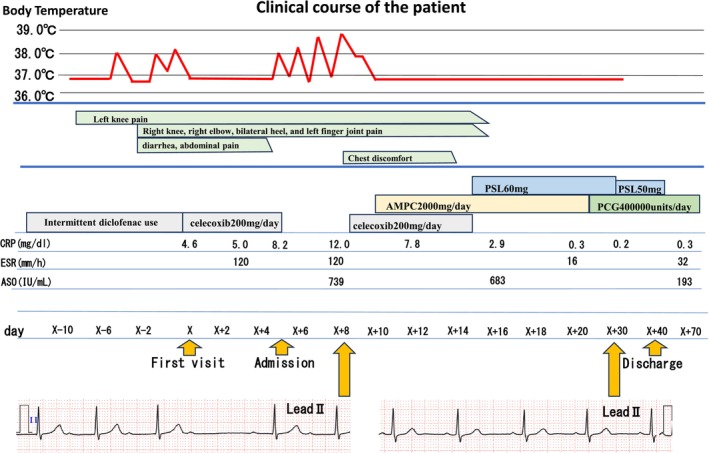
Clinical course of the patient. The figure shows the patient's clinical symptoms, body temperature, laboratory findings, treatments, and electrocardiographic changes during hospitalization. Lead II electrocardiograms obtained on day X + 13 (left) and before discharge (right) are shown. AMPC, amoxicillin; ASO, anti‐streptolysin O titer; CRP, C‐reactive protein; ESR, erythrocyte sedimentation rate; PCG, penicillin G; PSL, prednisolone.

The clinical course is shown in Figure 1. At the 1‐year follow‐up, the aortic regurgitation had resolved, while the mitral regurgitation remained stable without progression.

## 3 Discussion

Demonstrating preceding GAS infection is essential for the diagnosis of ARF. However, because ARF typically develops several weeks after the initial GAS infection, rapid antigen detection tests are often negative, and throat cultures are negative in approximately 75% of patients at the time of diagnosis. Furthermore, it is difficult to distinguish active infection from asymptomatic streptococcal carriage. Therefore, elevated *streptococcal* antibody titers (e.g., ASO) provide evidence of preceding GAS infection. However, an elevated ASO titer alone is insufficient for the diagnosis of ARF, which should be based on the overall clinical presentation together with the Jones criteria [1, 2, 3, 4].

This case was atypical for ARF because the arthritis was additive rather than migratory and was refractory to NSAIDs. Although additive arthritis is more characteristic of post‐*streptococcal* reactive arthritis (PSRA) than ARF [4, 5], PSRA is generally not associated with cardiac involvement [6]. Notably, a poor response to NSAIDs should prompt consideration of alternative diagnoses, even when ARF is suspected [4].

The differential diagnoses included infective endocarditis (IE), septic arthritis, juvenile idiopathic arthritis (JIA), systemic lupus erythematosus (SLE), adult‐onset Still's disease (AOSD), and sarcoidosis. IE was unlikely because repeated blood cultures were negative and no vegetations were detected on transthoracic or transesophageal echocardiography. Septic arthritis, SLE, AOSD, JIA, and sarcoidosis were considered unlikely because of the absence of joint effusion, negative antinuclear antibody testing, the absence of hyperferritinemia, hepatosplenomegaly, lymphadenopathy, or skin rash, the absence of hilar lymphadenopathy or elevated serum angiotensin‐converting enzyme activity, and the lack of characteristic findings on cardiac magnetic resonance imaging and gallium scintigraphy.

An observational study from India reported that 63% of patients with joint manifestations exhibited atypical articular features. Polyarthritis was frequently non‐migratory, and approximately 3% of patients showed a poor response to NSAIDs [7].

AV block is a recognized manifestation of ARF. While first‐degree AV block is generally attributed to increased vagal tone and is not considered evidence of carditis, Mobitz type II AV block is a rare complication (0.06%–2.6%) associated with carditis [8].

The WHO concluded that evidence is insufficient to recommend for or against corticosteroids because their effects on preventing RHD progression and reducing the severity of carditis or valvular lesions remain uncertain [9].

However, corticosteroids have been reported to improve carditis and atrioventricular block in patients with ARF [6, 10].

In the present case, corticosteroid therapy was initiated because of NSAID‐refractory Mobitz type II AV block, valvular involvement, and persistent arthritis. Guidelines recommend prednisolone 1–2 mg/kg/day (maximum 80 mg/day), tapered by 20%–25% weekly [6]. Because the patient weighed 120 kg, prednisolone was started at 60 mg/day. Mobitz type II AV block and joint pain resolved promptly.

Prednisolone was tapered without relapse, and aortic regurgitation resolved over the following months.

## 4 Conclusion

This case highlights that acute rheumatic fever should be considered even without a history of pharyngitis. Excessive NSAID use may mask fever and delay diagnosis. Therefore, careful assessment of prior analgesic use, cardiac findings, and *streptococcal* serology is essential in primary care.

## Author Contributions

**Fumiya Taketomi:** writing – review and editing. **Ikuma Noge:** writing – review and editing. **Hiroki Suzuyama:** writing – original draft, writing – review and editing. **Shigeki Nabeshima:** supervision. **Tadaaki Arimura:** writing – review and editing, validation. **Hiroaki Takeoka:** writing – review and editing. **Atsuhiko Sakamoto:** writing – review and editing. **Ryutaro Hashimoto:** writing – review and editing.

## Funding

The authors have nothing to report.

## Ethics Statement

Ethical approval was not required for this case report in accordance with institutional policy.

## Consent

Informed consent for publication was obtained from the patient.

## Conflicts of Interest

The authors declare no conflicts of interest.

## Data Availability

The data that support the findings of this study are available on request from the corresponding author. The data are not publicly available due to privacy or ethical restrictions.
